# Prevalence of appearance and weight dissatisfaction among female university students in France: Associations with BMI and body image avoidance

**DOI:** 10.1016/j.pmedr.2026.103680

**Published:** 2026-10-22

**Authors:** Axelle Nougal, Stéphane Rusinek, Thierry Kosinski

**Affiliations:** Univ. Lille, ULR 4072 - PSITEC - Psychologie : Interactions Temps Émotions Cognition, F-59000 Lille, France

**Keywords:** Body dissatisfaction, Body image avoidance, France, University, Women, Prevalence

## Abstract

**Objective:**

Body dissatisfaction has been widely studied among young women, but prevalence data in Europe remain scarce and no national prevalence estimates are available in France. Thus, this study aimed to estimate the prevalence of body dissatisfaction and examine body image avoidance among female university students in France.

**Methods:**

A total of 2092 French female students completed an online survey assessing appearance dissatisfaction, weight dissatisfaction, body image avoidance, body mass index (BMI) and sociodemographic variables in early 2026. Cut-off points were defined to estimate prevalence, and correlational analyses were conducted.

**Results:**

In our sample, up to 48.4% of participants were dissatisfied with their appearance, 45.3% with their weight, and 35.3% with both. Body image avoidance was strongly associated with both appearance and weight dissatisfaction.

**Conclusions:**

These findings highlight the need for easily disseminable public health interventions targeting body dissatisfaction and body image avoidance among university students, with the aim of reducing the risk of eating disorder onset, maintenance, and relapse.

## 1 Introduction

Body-image attitudes encompass individuals' perceptions, cognitions, affect, and behaviors regarding their physical attributes (Cash and Henry, 1995). The affective dimension, which includes satisfaction or dissatisfaction with one's body, is the dimension most commonly used to conceptualize body image (Fiske et al., 2014). Body dissatisfaction is one of the strongest predictors of the onset of eating disorders, and its prevalence varies according to several factors, including gender and age, with young women being among the most affected groups (Neighbors and Sobal, 2007; Stice et al., 2011). In addition, young women have been found to be among the populations with the highest prevalence of eating disorders (Silén and Keski-Rahkonen, 2022).

Body dissatisfaction is often associated with body image avoidance (e.g. avoiding reflective surfaces, hiding one's body with oversized clothing; Walker et al., 2018). According to the transdiagnostic model of eating disorders, body image avoidance is a behavioral expression of the overevaluation of shape, weight, eating and their control, which constitute the “core psychopathology” of eating disorders (Fairburn et al., 2003). Body image avoidance may reinforce body image concerns by preventing individuals from confronting the object of their concerns and potentially disconfirming their negative beliefs, thereby perpetuating body dissatisfaction and eating disorder pathology (Walker et al., 2018). Consistent with this, a meta-analysis of the relationships between body image avoidance and disordered eating has shown that body image avoidance is strongly positively associated with eating disorder pathology (Walker et al., 2018).

To date, no nationally representative study appears to have estimated the prevalence of body dissatisfaction in France. The only French study identified reported that 80.5% of young women in southern France wished to lose weight, providing an indirect indicator of body dissatisfaction in this population (Rodgers et al., 2010). In addition, body dissatisfaction appears to change over time at the societal level among women, with drive for thinness decreasing over time, whereas drive for muscularity appears to remain stable (Karazsia et al., 2016). Recent prevalence estimates using measures not limited to thinness-related dissatisfaction are therefore needed.

The present study aimed to estimate the prevalence of appearance and weight dissatisfaction among French female university students using the Body Esteem Scale for Adolescents and Adults (BESAA; Mendelson et al., 2001), as it is considered a relevant measure of body satisfaction that is not exclusively focused on thinness (Kling et al., 2019), and to examine associations with body image avoidance, BMI and parental socio-professional category. This study focused on young women given the particularly high prevalence of both body dissatisfaction and eating disorders observed in this population (Neighbors and Sobal, 2007; Silén and Keski-Rahkonen, 2022).

## 2 Method

### 2.1 Study design and population

A total of 2347 participants completed an online survey hosted on the LimeSurvey® platform in early 2026. Participants were excluded if they had missing data on the variables of interest or did not meet the inclusion criteria, namely being a woman, currently enrolled as a student, of French nationality, and being aged 18 to 30 years. As a result, 255 participants were excluded from analyses. The final analysis sample included a total of 2092 French female university students aged 18 to 30, with a mean age of 20.3 years (SD = 2.12). Participants were recruited from all regions of France and received no compensation. After providing informed consent, they completed sociodemographic questions and validated questionnaires assessing appearance satisfaction, weight satisfaction and body image avoidance. The study received ethical approval from the Research Ethics Board of the University of Lille and met the institution's guidelines for protection of human subjects concerning safety and privacy. Data is available online (see Appendix A).

### 2.2 Measures

Age, nationality, gender and student status were assessed to verify eligibility. Self-reported weight and height were used to calculate body mass index (BMI) and participants were classified according to the World Health Organization (WHO;, 1995) criteria. Parental socio-professional categories were assessed using the National Institute of Statistics and Economic Studies (INSEE;, 2024) categories and ranked according to median income for correlational analyses, based on INSEE (2025) data; with retirees, individuals without occupational activity, and missing data excluded from the analyses. Accordingly, 1716 participants were included in the analyses involving Parent 1, and 1659 in those involving Parent 2.

Appearance and weight satisfaction were assessed using the French version of the Appearance and Weight subscales of the Body Esteem Scale for Adolescents and Adults (BESAA; Mendelson et al., 2001; Rousseau et al., 2015). The subscales include 10 and 8 items, respectively, and consists of statements related to appearance and weight rated by respondents on a 5-point scale ranging from 0 (“never”) to 4 (“always”). Mean scores were computed for each subscale, with lower scores indicating greater dissatisfaction. Internal consistency was excellent for both subscales, α = 0.92 and α = 0.94 respectively.

Body image avoidance was assessed using the French version of the Body Image Avoidance Questionnaire (BIAQ; Maïano et al., 2009; Rosen et al., 1991). This questionnaire assesses body image avoidance across four dimensions: Clothing, Social Activities, Eating Restraint, and Grooming And Weighing. The 19 items are rated on a 6-point scale ranging from 0 (“never”) to 5 (“always”), with higher scores indicating greater body image avoidance. Internal consistency was acceptable (α = 0.77).

### 2.3 Statistical analysis

To estimate the prevalence of body dissatisfaction, in line with the recommendations of Fiske et al. (2014), two cut-off points were used, each applied to the Appearance and Weight subscale. First, following Cash and Henry (1995), scores below the neutral midpoint of 2.0 were classified as indicating dissatisfaction. Second, following Frederick et al. (2022), a more conservative cut-off of 1.75 was applied. Pearson's and Spearman's correlational analyses were conducted to examine associations between the study variables. All analyses were conducted using Jamovi (v. 2.7.39).

## 3 Results

Mean BMI was 22.4 kg/m^2^ (SD = 4.61). Descriptive statistics for body dissatisfaction and body image avoidance across BMI categories are presented in Table 1.

**Table 1:** Descriptive statistics of body satisfaction (appearance and weight) and body image avoidance across BMI categories among female students in France, January–March 2026.

	BMI				
	Underweight	Normal weight	Overweight	Obesity	
	(BMI < 18.5)	(BMI 18.5–24.9)	(BMI 25–29.9)	(BMI >30)	Total
	n = 280	n = 1411	n = 259	n = 142	n = 2092
*M (SD)*					
Appearance subscale	2.1 (0.8)	2 (0.8)	1.8 (0.8)	1.4 (0.8)	1.9 (0.8)
Weight subscale	2.4 (1)	2.2 (1)	1.3 (0.9)	0.8 (0.7)	2 (1)
Body Image Avoidance	29.6 (9.7)	30.5 (10.3)	35.1 (11)	39.6 (12.7)	31.6 (10.8)
					
*N (%)*					
Appearance subscale (<2)^a^	116 (41.4)	638 (45.3)	146 (56.3)	113 (79,6)	1013 (48.4)
(<1.75) ^b^	98 (35)	507 (35.9)	130 (50.2)	102 (71.8)	837 (40)
Weight subscale (<2)^a^	78 (27.9)	547 (38.7)	192 (74.1)	131 (92.2)	948 (45.3)
(<1.75) ^b^	66 (23.6)	448 (31.8)	169 (65.3)	127 (89.4)	810 (38.7)

BMI = Body Mass Index. Appearance and Weight subscales refer to the respective subscales of the Body Esteem Scale for Adolescents and Adults (BESAA).

^a^ Refers to participants whose mean score was strictly below the 2.0 midpoint on the 0–4 response scale.

^b^ Refers to participants whose mean score was strictly below the 1.75 cut-off point on the 0–4 response scale.

Depending on the cut-off used, 40.0% to 48.4% of participants were dissatisfied with their appearance, 38.7% to 45.3% with their weight, and 28.3% to 35.3% with both.

Correlational analyses are presented in Table 2. Body image avoidance was strongly negatively associated with weight satisfaction (*r* = −0.57, *p* < .001) and appearance satisfaction (*r* = −0.58, *p* < .001), and weakly positively correlated with BMI (*r* = 0.26, *p* < .001). BMI was weakly negatively correlated with appearance satisfaction (*r* = −0.22, *p* < .001) and moderately negatively correlated with weight satisfaction (*r* = −0.46, *p* < .001). Associations between parental socio-professional category and BMI, body satisfaction, or body image avoidance were very weak.

**Table 2:** Pearson and Spearman correlation matrix for BMI, body image avoidance, appearance and weight satisfaction and socio-professional category of parents among female university students in France, January–March 2026.

	BMI	Appearance subscale	Weight subscale	Body Image Avoidance	Socio-professsional category of parent 1	Socio-professional category of parent 2
BMI	–					
Appearance subscale	−0.22⁎⁎	–				
Weight subscale	−0.46⁎⁎	0.70⁎⁎	–			
Body Image Avoidance	0.26⁎⁎	−0.58⁎⁎	−0.57⁎⁎	–		
Socio-professional category of parent 1	−0.11⁎⁎	0.05⁎	0.07⁎	−0.04	–	
Socio-professional category of parent 2	−0.08⁎⁎	0.04	0.05⁎	−0.05⁎	0.40⁎⁎	–

The first four rows correspond to Pearson correlations and the last two rows correspond to Spearman correlations. BMI = Body Mass Index. Appearance and Weight subscales refer to the respective subscales of the Body Esteem Scale for Adolescents and Adults (BESAA).

⁎ *p* < .05.

⁎⁎ *p* < .001.

## 4 Discussion

This study aimed to estimate the prevalence of body dissatisfaction and examine body image avoidance among French female university students. Depending on the cut-off used, 40.0% to 48.4% of participants reported appearance dissatisfaction, 38.7% to 45.3% reported weight dissatisfaction, and 28.3% to 35.3% reported both.

As in previous research (Walker et al., 2018), body image avoidance was strongly associated with both appearance and weight dissatisfaction. This finding is consistent with the transdiagnostic model of eating disorders and the conceptualization of avoidance as a maintaining process of body dissatisfaction (Fairburn et al., 2003; Walker et al., 2018). It also suggests that prevention and intervention efforts should target not only negative body evaluation but also avoidance behaviors that may maintain distress and reinforce body-related concerns.

These findings are consistent with previous studies showing high levels of body dissatisfaction among women. However, prevalence in our sample was higher than that reported by Frederick et al. (2022), who found that 32% of U.S. women aged between 18 and 29 reported low appearance satisfaction and 31% reported high overweight preoccupation. This may partly reflect measurement differences, as the BESAA is not exclusively thinness-oriented and may capture broader forms of body dissatisfaction. Consistent with this interpretation, Fiske et al. (2014), in a review of the prevalence of body dissatisfaction in the United States, noted that prevalence estimates vary substantially depending on the measure used. For example, a large German study using a global single-item measure not exclusively thinness-oriented found a prevalence of 48.3% of weight dissatisfaction among their participants, which is comparable to the prevalence observed in our study (von Lengerke et al., 2012). Overall, our findings confirm that body dissatisfaction is highly prevalent among French female university students. This high prevalence can be partly explained by factors such as a greater appearance investment, self-objectification, and internalization of thin and fit ideals in this population (Dane and Bhatia, 2023).

Consistent with previous research, BMI was significantly associated with body dissatisfaction (Rodgers et al., 2010). However, the associations observed here were weak to moderate, suggesting that appearance dissatisfaction is likely shaped by a broader range of factors. In contrast, parental socio-professional category showed only very weak associations with body dissatisfaction and avoidance, suggesting a limited role in this population.

This study has several limitations. First, the cut-off points used to estimate prevalence were derived from previous studies but have not been formally validated for the BESAA. Second, BMI was calculated based on self-reported height and weight. Third, the study assessed overall appearance and weight dissatisfaction but did not examine specific forms of dissatisfaction, such as thinness-, muscularity-, or fit-oriented concerns (Karazsia et al., 2016). Finally, the study was restricted to female university students and did not assess the prevalence of body dissatisfaction among male students or genders minorities.

Despite these limitations, this study provides the first large-scale estimate of appearance and weight dissatisfaction among female university students in France. Its main strengths include its adherence to the recommendations proposed by Fiske et al. (2014) for prevalence studies on body dissatisfaction, particularly the use of a large national sample (*n* > 800), the use of internationally recognized measures and the application of multiple cut-off points. Given the focus on female students in the present study, future research should examine body dissatisfaction among male students to enable comparisons with prevalence estimates among young women in France and male students in other countries, as body dissatisfaction remains prevalent among young men (Frederick et al., 2022).

## 5 Conclusion

Overall, our findings indicate that body dissatisfaction is highly prevalent among female university students in France and that body image avoidance is also elevated in this population as nearly half of participants reported some form of body dissatisfaction, and body dissatisfaction was strongly associated with body image avoidance. Because both constructs have been identified as central processes in the onset, maintenance, and relapse of eating disorders, these findings underscore the importance of developing interventions that can be widely disseminated and that directly target both body dissatisfaction and body image avoidance with the aim of reducing eating disorder risk (Fairburn et al., 2003; Stice et al., 2011).

## CRediT authorship contribution statement

**Axelle Nougal:** Writing – review & editing, Writing – original draft, Methodology, Investigation, Funding acquisition, Formal analysis, Conceptualization, Visualization. **Stéphane Rusinek:** Writing – review & editing, Validation, Supervision, Funding acquisition, Conceptualization, Project administration. **Thierry Kosinski:** Writing – review & editing, Validation, Supervision, Project administration, Methodology.

## Ethics declaration

### Informed consent and patient details

Written informed consent to take part in the study and to publish the article has been obtained from all participants or their legal representatives. The privacy rights of participants have been observed.

### Studies in human

This study was performed in compliance with relevant laws, regulatory frameworks and guidelines where the research took place. This study was approved by the Research Ethics Board of the University of Lille. (Approval No. 2025-956-S142).

## Declaration of competing interest

The authors declare that they have no known competing financial interests or personal relationships that could have appeared to influence the work reported in this paper.

## Data Availability

The dataset associated with this study is available online at the following link: https://osf.io/qbfyu/overview?view_only=0fcfe036977b441dbd05a3f8c3b80729
